# Improving Blood Pressure Control Through Standardization of Workflow in Outpatient Internal Medicine Clinics

**DOI:** 10.1016/j.jaccas.2025.105662

**Published:** 2025-10-07

**Authors:** Taysir Al Janabi, Sulihat Mudasiru, FNU Naintara, Chrysanthi Lundstedt, Taha Abdul Rehman, Abdul Raheem, Andrew Amparo

**Affiliations:** Department of Internal Medicine, WellSpan York Hospital, York, Pennsylvania, USA

**Keywords:** blood pressure control, hypertension, quality improvement, WellSpan health, WellSpan York Hospital, workflow standardization

## Abstract

**Background:**

Uncontrolled hypertension (HTN) affected billions of people worldwide and significantly increased the risk of cardiovascular disease-related mortality. Reviewing blood pressure (BP) data from WellSpan internal medicine clinics patients revealed an opportunity for improvement.

**Project Rationale:**

The project intent was to improve BP control in patients with BP ≥140/90 mm Hg and achieve 85% HTN control by June 30, 2025.

**Project Summary:**

The HTN Champion Initiative was a resident-led program that successfully established a workflow for stricter BP control through collaboration among residents, staff, and patients, with a focus on skill acquisition and education.

**Take-Home Messages:**

The HTN Champion Initiative had a meaningful impact on BP control in our clinic patient panel. Although the quality metric of our project was BP control in real time, the expected downstream effect was the associated long-term mortality benefit.

## Graphical Abstract

**Figure:**
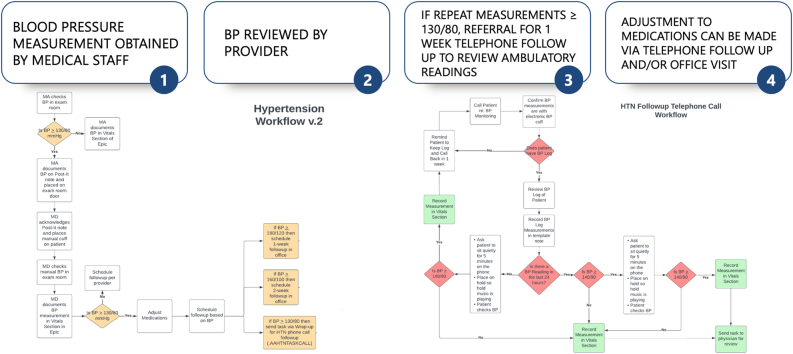

According to the Centers for Disease Control and Prevention, hypertension (HTN) had been the primary cause of 685,875 deaths in the United States in 2022. Approximately 48.1% of Americans who had received a HTN diagnosis were not taking blood pressure (BP)-lowering medications. However, about 34 million hypertensive adults in the U.S. had not started an antihypertensive agent.^1^ According to a systematic review published in 2018 regarding the association of BP and mortality, antihypertensive treatment was only associated with a reduced risk of death and major adverse cardiac events if systolic BP was 140 mm Hg or above.^2^ A national study on HTN prevalence in the U.S. from 2017 to 2018 found that 39.64% of hypertensive individuals taking medication had well-controlled BP.^3^ Although the WellSpan internal medicine clinic HTN control rates remained above than the national average, a notable percentage of patients could have benefited from stricter BP control in terms of mortality.

**Take-Home Messages**

• HTN workflow standardization had streamlined HTN management.
• The HTN Champion Initiative had made a meaningful impact through education and skill development.

In the electronic medical record system, EPIC, BP was designated as “controlled” if the most recently entered BP measurement at any WellSpan Health office was <140/90 mm Hg. Patients with measurements ≥140/90 mm Hg or those who had not had a measurement within the past year would not satisfy this condition. HTN control at the WellSpan internal medicine clinics had been below the Bluebook Objective set by WellSpan Health in 2021-2022. This HTN Champion Initiative aimed to improve HTN control at WellSpan internal medicine clinics and achieve 85% BP control by the end of June 2025.

This article outlined the planning, execution, and results of the quality improvement (QI) project, detailing the key interventions that drove change. This project provided a practical framework for other primary care settings aiming to optimize BP control and minimize cardiovascular risk among their patients.

## Case Summary Prompting the Project Launch

BP control rate at WellSpan internal medicine clinics had been below the WellSpan Bluebook Objective; the HTN control in 2021-2022 was low at 72.74% while the Bluebook Objective for HTN control for that year was at 77.26%. This gap created an opportunity to improve cardiovascular outcomes for a significant portion of our patients through education. In addition, it allowed residents to improve communication, coordination, and collaboration between patients and clinic staff.

## Project Rationale

Medical residents, with their inherent leadership potential, were well-suited for planning and executing health projects. This QI project was designed to leverage the leadership and communication skills of medical residents in improving BP control at WellSpan internal medicine clinics. By actively reaching out to individual residents and providing 1-on-1 technical assistance, medical residents were able to offer an evidence-based and personalized approach for their patients in addressing HTN. In addition, this QI project assisted medical residents in honing their communication and leadership skills. The rationale of this project was based on the evidence of mortality benefits from BP optimization.

## Project Description

A new committee of residents (3 postgraduate year-3 residents, 2 postgraduation year-2 residents, and 1 intern) had been established to collect, review, and analyze data from EPIC. An initial analysis showed the percentage of hypertensive patients whose BP had been controlled was below the WellSpan Bluebook Objective of 2021 to 2022 of at least 77.26%. Data were collected from EPIC and through a survey of residents and clinic staff. Areas for improvement were identified, including recommendations for rechecking BP during visits, follow-up, communication among clinic staff, and improvements in looking up uncontrolled HTN patients on EPIC.

**Figure 1:**
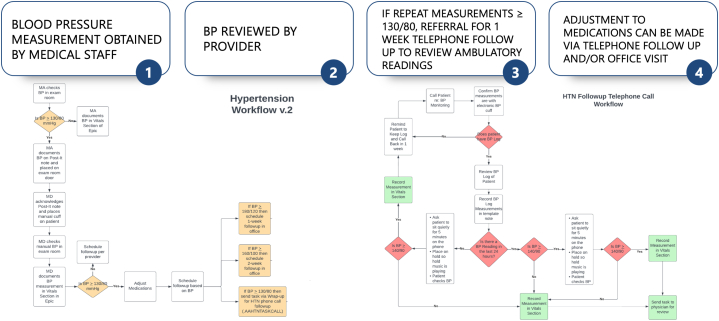
The Initial Standardized HTN QI Workflow The standardized workflows outline the necessary steps of checking, rechecking, and communicating with clinic staff on elevated blood pressure readings. HTN = hypertension; QI = quality improvement.

The committee developed workflows that established a protocol on how to check, recheck, and educate patients on how to check their BP at home (Figure 1). In addition, a smart phrase was created to forward follow-up requests to a designated staff person who would contact patients in a week to obtain their home BP readings; this QI project team developed a BP log that residents or clinic staff handed out to patients with elevated BP. Patients were instructed to check their BP at least once daily for 1 week and document their BP reading using the log. At the end of the week, a designated clinic staff member would call the patient to obtain these numbers. In addition, patients could also send the log using the patients' messaging portal via EPIC. Moreover, patients could also drop off the log at the office.

Residents, under the supervision of their faculty or senior residents, provided guidance on ensuring patients' compliance, checking the accuracy of BP measurement, understanding the situation under which BP was measured, and adjusting antihypertension medications. For example, several patients who were labeled as having uncontrolled BP had their BP taken during a urology or orthopedic urgent care visit where they were receiving intraarticular injections for pain, which might have contributed to their elevated BP.

**Figure 2:**
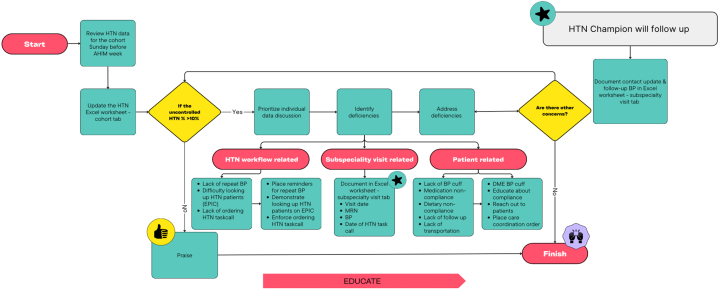
The HTN Champion Initiative Workflow The HTN Champion Initiative workflow outlines the key components and the leadership role of HTN champions.

These initiatives had improved HTN control in meeting the WellSpan Bluebook Objective for 2023 to 2024 of 79.08%. However, a new initiative was established to address challenges in increasing HTN control to 85%. The WellSpan York's internal medicine residency program had 5 cohorts, each consisting of 6 to 7 residents who rotated at the clinic every 5 weeks. A self-motivated resident from each cohort was selected to be part of the new initiative, known as the HTN Champion Initiative (Figure 2). The role of the HTN Champion was to act as a leader within their cohort, identifying residents whose uncontrolled HTN patients equaled or exceeded 10% and providing them support and guidance. In addition, a database was established to track those with elevated BP at subspecialty clinics. A designated HTN Champion would follow up on those patients.

## Project Deliverables

**Figure 3:**
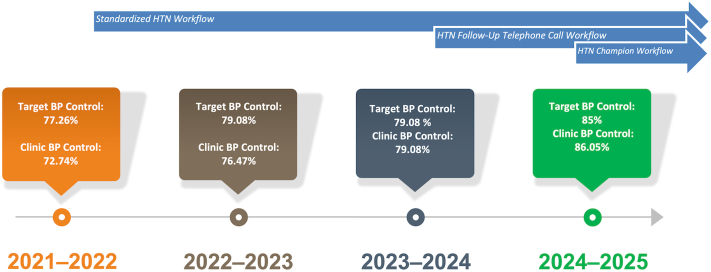
The Timeline of the Implementation and Impact of Different Hypertension Workflows The standardized hypertension (HTN) workflow was implemented in 2021 to 2022. The HTN follow-up telephone call workflow was executed in 2023 to 2024. The HTN Champion Initiative workflow was implemented in 2024 to 2025.

This project had several deliverables, including workflows, BP logs, reminders, a HTN smart phrase, and a database to track individuals whose BP had been elevated at subspecialty clinics. Workflows included the standardized HTN workflow, which had established a standard process for checking, rechecking, and communicating with clinic staff. In addition, the HTN follow-up telephone call workflow and the HTN Champion Initiative were 2 protocols that had been added later (Figure 3).

## Project Outcome, Impact, and Future Directions/Next Steps

**Figure 4:**
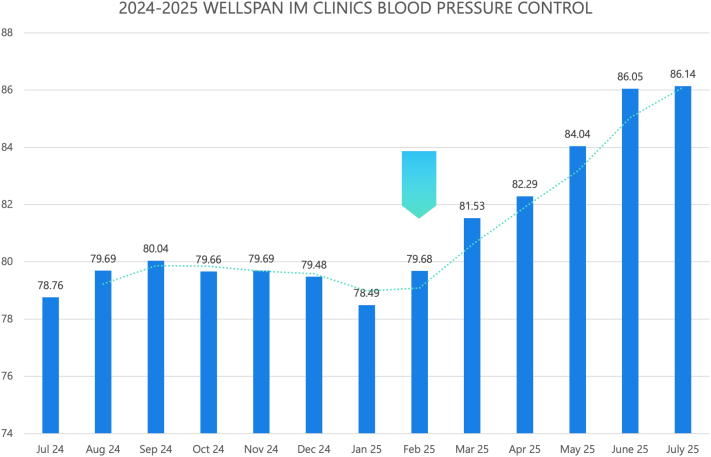
The Impact of the HTN Champion Initiative Workflow The arrow represents the time when the HTN Champion Initiative was implemented.

The outcome of this QI project was to improve HTN control at WellSpan internal medicine clinics, as measured by the percentage of patients with well-controlled BP. The initial workflows enabled the team to achieve the target BP control rate of 77.26%. Based on lessons learned from initial workflows, the HTN Champion Initiative workflow was developed, which improved HTN control to 86.05% in 4 months (Figure 4). By the end of June 2025, the total number of hypertensive individuals was 2,587. Of those, 2,226 (86.05%) had well-controlled BP in our clinics. Out of the individuals with uncontrolled HTN (361), 162 (44.8%) had a documented elevated BP at different subspecialty visits, requiring follow-up.

The next step for this QI project was to sustain the short-term meaningful impact of the HTN Champion Initiative. The development of a medication protocol was also under consideration as part of the next steps for this QI project. In addition, achieving the new BP control target for 2025 to 2026, a target that had yet to be released, would be another goal for this project. Moreover, new health policies were to be established to optimize BP control.

## Discussion

In a community-based primary care clinic, staffed by internal medicine residents under faculty supervision, implementing a standardized HTN protocol was associated with a 14% improvement in HTN control, exceeding the target set by our institution; 6% of this improvement was achieved within 4 months of implementing the HTN Champion Initiative.

Several key process changes can be attributed to this success. First, conducting a survey of our residents and clinic staff allowed us to identify challenges that resulted in lower HTN control in our clinics, including time constraints, a lack of follow-up with patients with documented elevated BP during office visits, a lack of communication between residents and clinic staff, and a lack of knowledge among residents on tracking their uncontrolled HTN numbers in EPIC. Shortly afterward, the standardized HTN protocol was created, directly addressing the barriers identified earlier. The impact of the protocol was to decrease variation in BP management of hypertensive patients in our clinic.

In addition, providing clear details on next steps for residents lowered the threshold to intensification of therapy by residents. A similar approach was implemented by Jaffe et al^4^ in their Kaiser Permanente Northern California Hypertension Program, which involved implementation of a single-pill combination antihypertensive in an attempt to decrease variations in their HTN program.

Another key observation was that the standardized workflow facilitated frequent reassessment of patients' home BP readings. This enhanced surveillance allowed earlier recognition of uncontrolled HTN and likely contributed to the observed improvements in HTN control. Shantharam et al documented that frequent BP monitoring coupled with clinician feedback is a known mechanism for improving HTN.^5^

Although our standardized HTN workflow improved and met the target set by WellSpan Bluebook Objectives, we continued to experience challenges with protocol adherence among clinic residents. Champions identify residents whose uncontrolled HTN percentage equals at least 10%, and they prioritize providing feedback to those residents and reinforcing adherence to the HTN protocol. The impact of this approach has fostered a culture of accountability in our clinics and incentivized residents to enhance HTN control within their patient panels.

## Conclusions

This QI project had shown that workflow standardization had a meaningful impact on BP control at WellSpan internal medicine clinics. In addition, reviewing data, surveying residents, and communicating with staff had helped identify areas for improvement and develop interventions accordingly. The HTN Champion Initiative had improved BP control by assigning a leader within each cohort to identify deficiencies, intervene, and assess progress. This QI project could be replicated by other academic institutions where HTN control was not optimal.

## Funding Support and Author Disclosures

The authors have reported that they have no relationships relevant to the contents of this paper to disclose.
